# Mfn2 inhibits proliferation and cell-cycle in Hela cells via Ras-NF-κB signal pathway

**DOI:** 10.1186/s12935-019-0916-9

**Published:** 2019-07-29

**Authors:** Xiaowen Liu, Jun Sun, Ping Yuan, Kangquan Shou, Yuanhong Zhou, Wenqi Gao, Jin She, Jun Hu, Jun Yang, Jian Yang

**Affiliations:** 1 Hubei Key Laboratory of Tumor Microenvironment and Immunotherapy, Medical College, China Three Gorges University, Yichang, China; 2 Institute of Cardiovascular Research & Department of Center Experiment Laboratory, the First College of Clinical Medical Science, China Three Gorges University, Yichang, 44300 China; 3 Department of Biochemistry and Molecular Biology, Basic Medical College, Tongji Medical College, Huazhong University of Science and Technology, Wuhan, China

**Keywords:** Mfn2, Ras, Cervical carcinoma, Cell proliferation, Cell-cycle

## Abstract

**Background:**

Mitofusin 2 (Mfn2) is outer membrane protein, as the inhibitor of Ras protein. This study aimed to investigate the effect of Mfn2 on cell proliferation, and cell-cycle in Hela cervical carcinoma cell lines.

**Methods:**

After treated with Adv-mfn2 or Adv-control for 48 h and 60 h, the RNA and protein of Mfn2 in Hela cells were detected by qRT-PCR and western blot. The immunofluorescence assay was performed to observe the expression and sub-location of Mfn2 in Hela cells. The flow cytometry was performed to detect the cell cycle of Hela cells, while western blots were performed to observe the Ras-NF-κB signal pathway. Then, the xenografted cervix carcinoma mouse model was used to confirm the effect of Mfn2 in Hela cells in vivo and the expression of Ras-NF-κB signaling pathway in vivo.

**Results:**

In immunofluorescence detection, Mfn2 was located in cytoplasmic, not in the nucleus. In addition, Mfn2 inhibited cell proliferation of Hela cells through reducing PCNA protein expression. Mfn2 induced arrest in G0/G1 phase of the cell cycle in Hela cells. Meanwhile, Mfn2 reduced Cyclin D1 protein expression. Moreover, Mfn2 decreased the Ras signal pathway proteins such as Myc, NF-κB p65, STAT3 in a dose-dependent manner. Then, the in vivo experiment also confirmed that Mfn2 could inhibit the tumor growth, and depress the Cyclin D1, Ras, Myc, NF-κB p65, Erk1/2 and mTOR protein expression.

**Conclusions:**

Mfn2 could significantly inhibit cell proliferation in Hela cells. It might be acted as an potential anti-cancer target through inducing cell cycle arrest in human cervical carcinoma cells.

## Background

Cell hyper-proliferation has long been considered as an important etiological factor of many cancers, and self-sufficiency proliferation signals are sustaining, such as Ras signal pathway [1, 2]. Mitofusin 2 (Mfn2) is involved in regulation of cell survival and has been of interest in cancer field [3–6]. It may be involved in cervical cancer pathogenesis and might serve as a biomarker of cervical SCC in the future [7]. In our and others studies, Mfn2 could induce Hela cells into mitochondrial apoptosis [8], pancreatic cancer into cell autophagy [9], and breast cancer into DNA methylation [10], as a tumour suppressor in many cancers. Mfn-2 is a mitochondrial outer-membrane protein with GTPase activity involved in mitochondrial fusion and fission and apoptosis regulation [11–13]. *Mfn2* gene encodes for a 757-amino-acid protein containing a p21Ras domains near the N-ter [14]. The p21Ras domain makes Mfn2 as an anti-Ras protein in VSMCs, and regulating the VSMCs cell-cycle [15]. In human breast cancer, overexpression of Mfn2 inhibited the Ras-ERK1/2 signaling pathway, but with deletion of the p21Ras motif partially reduced the anti-tumor function of Mfn2 [10].

In our early studies, we found that PTD4-apoptin fusion protein could upgrade Mfn2 expression in cervical carcinoma cells [16]. Then, we found that when the expression of Mfn2 increased, the Hela cells were induced into apoptosis via mitochondrial pathway [8]. In this study, we aimed to investigate whether Mfn2 was involved in proliferation in Hela cells, and activated Ras signaling pathways to inhibit Hela cells proliferation. Our findings provide a new target of cervical carcinoma and suggest candidates for potential use in cervical carcinoma therapy in the future.

## Materials and methods

### Antibodies

Antibodies were as follows: Anti-Mfn2 (D2D10) (Cell Signaling, 9482) directed against Mfn2 protein, Ras (Cell Signaling, 3965), Cyclin D1 (Cell Signaling, 2922), p44/42 MAPK (Erk1/2, Cell Signaling, 9102), PCNA (PC10) (Cell Signaling, 2586), Myc (Cell Signaling, 5605), mTOR (Cell Signaling, 2972), STAT3 (Cell Signaling, 9139) and NF-κB p65 GAPDH (G-9) (Santa Cruz, sc-365062, monoclonal, mouse) and β-actin (Tianjin Sungene Biotech, China) antibodies were used as the loading controls.

### Construction of mfn2 expression adenoviral vector

Rno-mfn2 precursor DNA (*Homo sa*piens (human), Gene ID: 9927) was synthesized by Genechem (Shanghai, China). The adenovirus expressing mfn2 (Adv-mfn2), or control adenovirus expressing control (Adv-control) was generated using the AdMax system (Microbix Biosystems, Canada) according to Wang’s in 2018 [8].

### Cell culture

The human cervical carcinoma cell line HeLa was purchased from the Chinese Culture Tissue Collection Center (CCTCC, China). The cells were cultured in DMEM (Hyclone, USA) supplemented with 10% FBS (fetal bovine serum; Hyclone, USA) at 37 °C and 5% CO_2_.

### qRT-PCR analysis

The total RNA from Hela cells was extracted using TRIzol@ Reagent (Invitrogen, USA). Reverse transcription and qRT-PCR were performed as described previously [8, 17, 18]. Amplification and detection of specific products were performed with the ABI stepone plus (PE Applied Biosystems). The mfn2 mRNA expression was measured by RevertAid Reverse Transcriptase (Thermo scientific, EP0442) and qPCR Master Mix (Fermentas,K0221), and the GAPDH was used as an internal control. The 2−ΔΔCt method was used to measure the realtime PCR Data. The following sequence-specific primers of Mfn2 were as follow: F: 5′-ATCTGTGCCAGCAAGTTGACA-3′ and R: 5′-AAGTGAATCCAGAGCCTCGAC-3′.

### CCK-8 test

The cells, seeded into the 96-well plate at 3000 cells per well, were incubated with 50, 100 and 150 pfu/cell of Mfn2 or as negative control with PBS for 48 and 60 h in 5% CO_2_ at 37 °C according to Wang’s paper in 2018 [8].

### Cell cycle

The cells, seeded into the 6-well plate at 1 × 10^5^ cells per well, were incubated with 100 pfu/cell Adv-mfn2 or Adv-control of Adv-mfn2 or Adv-control for 60 h in 5% CO_2_ at 37 °C. Cells were then washed twice in ice-cold PBS, stained with Cell Cycle and Apoptosis Analysis Kit (Beyotime, C1052, China), for 30 min at room temperature and analyzed with a BD FACSort flow cytometer (BD Biosciences, USA). Cell cycle data was analyzed by ModFit LT 3.2 software (Verity Software House, Topsham, USA).

### Immunofluorescence

HeLa cells were seeded in 6-well plates at a ratio of 10,000 cells per well. After 12 h, Adv-mfn2 or Adv-control was added into the medium of 50 pfu/cell and incubated at 37 °C. After 60 h, the cells were washed 3 times with PBS and fixed with 4% paraformaldehyde for 10 min at room temperature. Subsequently, the cells were washed 3 times with PBS and permeabilized for 5 min with PBS containing 0.2% Triton X-100. Anti-mfn2 antibodies were used to detect the presence and cellular localization of mfn2 protein in HeLa cells, as recently reported [16]. The appropriate Rhodamine-conjugated goat anti-mouse IgG antibodies (Pierce, 31569) were used as secondary antibodies. The cellular nuclei were stained with 4, 6-diamidino-2-phenylindole (DAPI, 1 μg/L in PBS, Roche, 10236276001). The cells were analyzed by means of confocal fluorescence microscopy Fluoview FV50 (Olympus, Japan).

### Western blotting

The Whole cell proteins and tissue proteins from model mouse were extracted by ice-cold SDS lysis buffer. The BCA protein assay kit (Pierce, 23227) was used to determine the protein concentrations according to the manufacturer’s instructions. The proteins were fractionated on 12% and 15% SDS-polyacrylamide gel and electroblotted onto Immobilon-P PVDF transfer membranes (Millipore, IPVH08130), as recently reported [8]. The blots were incubated with Mfn-2, anti-Ras, Cyclin D1, ERK1/2, PCNA, c-Myc, mTOR, STAT3 and NF-κB p65. GAPDH and β-actin were used as the loading controls. The positive signals were visualized by Odyssey^®^ Two-Color Infrared Imaging System (Li-Cor, USA).

### Xenografted cervix carcinoma mouse model

BALB/c nude mice (4–5 weeks old, female) were obtained from the Hubei Provincial Center for Disease Control and Prevention (HBCDC, China). 5 × 10^5^ Human cervix carcinoma HeLa cells were collected and injected subcutaneously under alar of nude mice as recently reported [8]. When the tumors were visible, the mice were divided randomly into 2 groups, consisting of 5 tumor-bearing mice per group for a 2-week-treatment with Adv-mfn2 or Adv-control, respectively. The solution samples were infected into the tumor tissue. Every 3 day 5×10^8^ pfu/ml Adv-mfn2 or Adv-control were applied per mouse as our last paper [8]. All animal studies were carried out in accordance with the “Guide for the Care and Use of Laboratory Animals” and approved by the Hubei Provincial Center for Disease Control and Prevention (HBCDC, China).

### Detection of xenografted tumor growth

The tumor volume was measured before and after the 2-week-treatment by the following formula: volume = 0.52 × length × width^2^ [8, 16]. The tumor volume difference was calculated by the following formula: difference = after volume – before volume. After 2-week-treatment, the tumor tissues were obtained, and prepared for HE stains.

### HE assay

After a 2-week-treatment, respectively, the mice were killed and the tumors were fixed with 4% paraformaldehyde, and paraffin sections were prepared for carrying out HE assay, as recently reported [17].

### Statistics

The Student’s t test was used to determine the statistical significance of data. A p value of less than 0.05 (*) or less than 0.01 (**) was considered to be significant. Data presented in the figures represent the mean ± standard error.

### Ethics statement

Our animal studies were carried out with the Guide for the Care and Use of Laboratory Animals of the People’s Republic of China in strict compliance. All efforts were made to minimize suffering and all procedures were performed under ethylether anesthesia. The study protocol was approved by the Committee on the Ethics of Animal Experiments of the Chinese Centre for Diseases Control and Prevention.

## Results

### Over-expression of Mfn2 in Hela cells

**Fig. 1:**
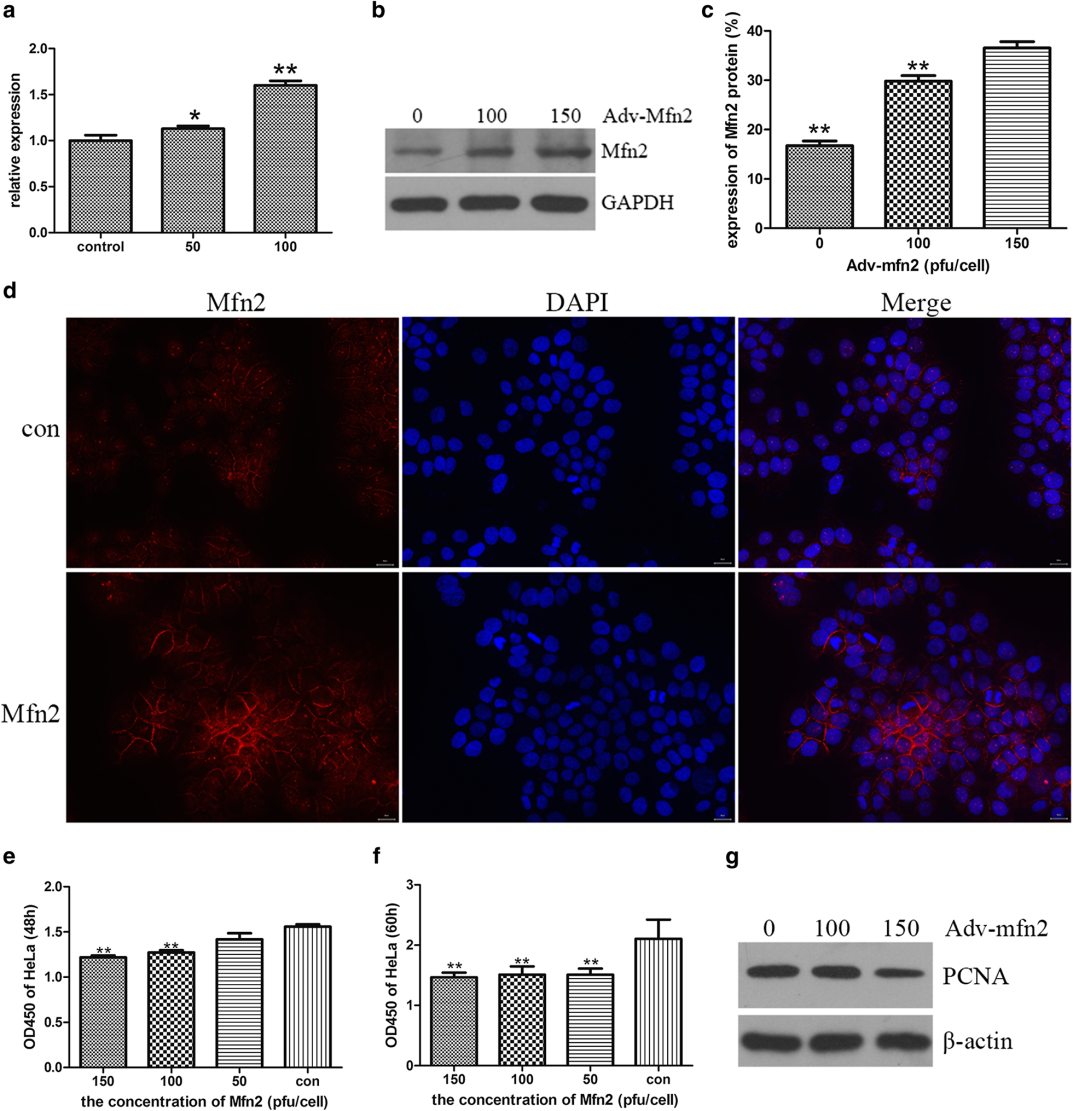
The overexpression of Mfn2 and its effect on cell proliferation in Hela cells. **a** HeLa cells were incubated with Ad-Mfn2 (100 or 150pfu/cell) or Adv-control. The expression of Mfn2 mRNA in the Hela cells was detected by qRT-PCR. Data are represented as mean ± SD (*p < 0.05, **p < 0.01). **b**, **c** Cell lysate was extracted from HeLa cells treated with Adv-control, Adv-Mfn2 (100 or 150pfu/cell) for Mfn2 expression by western blot analysis (**b**) and quantitation (**c**). GAPDH was used as reference. **d** Adv-mfn2 were added to the medium of the cells, and incubated for 60 h. Cellular staining of nuclear DNA (DAPI) and localization of Mfn2 protein in HeLa cells. Magnification: 200×. **e**, **f** HeLa cells were exposed to different concentrations of Adv-Mfn2 (0, 50, 100, 150 pfu/cell) for 48 h and 60 h, and then the cell viability was measured by CCK-8 assay. The Hela cells decreased after being treated with Adv-Mfn2 in a dose- and time-dependent manner. (mean ± SD from three independent experiments, **p < 0.01). **g** After incubated with Adv-mfn2 (0, 100 and 150 pfu/cell), the expression of PCNA protein in HeLa cells was detected by western blot. β-actin was used as reference

To detect whether the adenovirus took the mfn2 gene into the cervix carcinoma cells, we uesd the qRT-PCR to obverse the expression of mfn2 gene in Hela cells. Adv-mfn2 or Adv-control was given into the wells with 1 × 10^6^ Hela cells. After incubated for 60 h, the cells were collected, and lysed in ice-cold TRIzol@ Reagent to obtain the total RNA. Figure 1a shows that the adenovirus take the mfn2 gene into the cells, and increases the expression of mfn2 gene. Then, we detected the expression of Mfn2 protein in Hela cells. We dissolved the Hela cells which were incubated with Adv-mfn2 or Adv-control for 60 h, and used the western blot to obverse the expression of Mfn2 proteins. We could see that Mfn2 proteins were over-expression in Adv-mfn2 Hela cells (Fig. 1b, c).

### Cellular location of Mfn2 in Hela cells

Mfn2 is considered as the outer membrane protein of the mitochondria [11], so we analyzed the cellular location of Mfn2 in Hela cells. The Hela cells were incubated with 50 pfu/cell Adv-mfn2 or Adv-control for 60 h in 6-well plate at 37 °C. Then, the Mfn2 antibody was incubated in the wells, as DAPI for cellular nuclear. Across the fluorescence microscope, we examined the cellular location of Mfn2 in Hela cells (Fig. 1d). The Mfn2 proteins were increased in Mfn2 group, and located in the cytoplasmic, not in the nuclear.

### Mfn2 inhibits the growth of cervix carcinoma cells

The CCK-8 was performed for detecting the relative inhibition rate of Hela cells. To obverse the inhibition of Mfn2 in Hela cells, we set the CCK-8 to detect the relative inhibition rate of Hela cells. The cells were seeded into the 96-well plates as 3000 cells per well. After 12 h, the mediums of 50, 100 and 150 pfu/cell of Adv-mfn2 or Adv-control was incubated into the wells. After 0, 48 and 60 h, the cells were analyzed by CCK-8 test. We could found that the relative inhibition of Mfn2 in Hela increased, depend on the time and the dose (Fig. 1e, f). 60 h incubation group had the highest inhibition, as well as the 150 pfu/cell Adv-mfn2. It was suggested that Mfn2 could inhibit Hela cells growth with time and dose dependence.

Proliferating Cell Nuclear Antigen (PCNA) is the major coordinator of faithful and processive replication and DNA repair at replication forks [19]. It is bound up with the cells proliferation. Here, PCNA protein was examined to proof the inhibition of Mfn2 in Hela cells. The expression of PCNA in Mfn2 groups was decreased obviously in western blot result in Fig. 1g dependence on the dose of Mfn2.

### Over-expression of Mfn2 altered cell-cycle in Hela cells

**Fig. 2:**
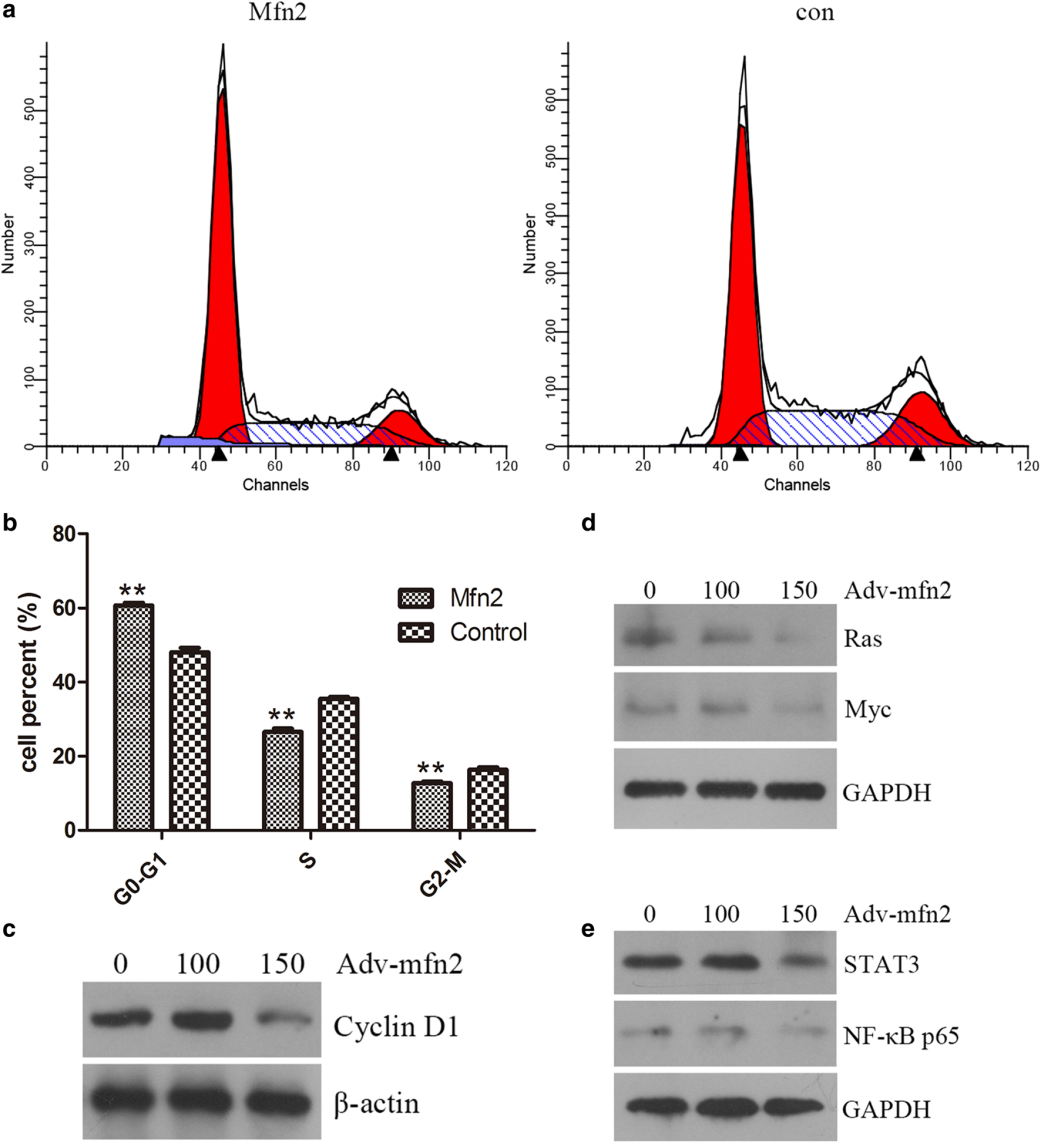
The effect and the signal pathway of Mfn2 on cell-cycle in Hela cells. **a** HeLa cells were incubated with Ad-Mfn2 for 60 h. Cells stained with Cell-Cycle assay after incubated with two concentrations (100 pfu/cell) of Adv-Mfn2 were measured by flow cytometry. **b** The percentage of every phase of cell-cycle after HeLa cells being treated with indicated concentrations of Adv-Mfn2 or Adv-control was calculated. **c** Adv-Mfn2 increased the expression of Cyclin D1 by western blot. Beta-actin was used as reference. **d** Expressions of Ras and Myc after HeLa cells being treated with Adv-Mfn2. GAPDH was used as reference. **e** Expressions of STAT3 and NF-κB after HeLa cells being treated with Adv-Mfn2. GAPDH was used as reference

To confirm the possible mechanism of inhibition of Mfn2 in Hela cells, the cell-cycle test was performed in Hela cells incubated with Adv-mfn2. The 100 pfu/cell of Adv-mfn2 or Adv-control was added into Hela cells in 6-well plate for 60 h. Fluorescence activated cell sorting (FACS) analysis was used to examine cell-cycle distribution. Adv-control added into well of Hela cells, approximately 35.53% of Hela cells infected by Adv-control progressed into S phase. On the contrary, Hela cells infected with Adv-mfn2 remained mostly in the G0/G1 phases with only 26.58% of cells entering S phase. We speculated that Mfn2 could further block the Hela cells in G0/G1 phase due to the effects of Adv-mfn2 on Hela cells proliferation as shown in Fig. 2a, b.

Cyclin D1 is an oncogene frequently overexpressed in human cancers that has a dual function as cell cycle and transcriptional regulator, although the latter is widely unexplored [18]. Western Blot of Cyclin D1 was used to further confirm that the cell-cycle of Hela cells was inhibited by Cyclin D1 down-expression in Hela cells, depend on the dose of Mfn2 in Fig. 2c.

### Mfn2 activity is affected via Ras signal pathway

The function of the RAS signaling pathway is to integrate extracellular signals and coordinate a suitable response by a subsequent control of cellular growth, survival, and differentiation [20, 21]. Mfn-2 is known to block cell proliferation via inhibition of the Ras pathway in VSMCs [15, 22]. We examined whether Mfn2-mediated inhibition of cell proliferation and cell cycle in Hela cells was affected by the cellular level of Ras. HeLa cells were incubated with Adv-mfn2 or Adv-control. The results represented in Fig. 2d demonstrate that the expression of Ras are affected by Mfn2. Over-expression of Mfn2 could increase the Ras and Myc protein expression in HeLa cells. Meanwhile, the expressions of STAT3 and NF-κB p65 protein were also decreased by incubation with Adv-Mfn2 (Fig. 2d, e).

Therefore, we conclude that Mfn2 inhibits Hele cells proliferation and cell-cycle by activating Ras protein expression. Furthermore, our finding that Myc, STAT3 and NF-κB p65 protein are decreased by incubated with Adv-mfn2 in HeLa cells. The present research showed that Ras was sensitive in Mfn2-induced proliferation depressing in HeLa cells, and inactivated its downstream signal pathway as Myc, STAT3 and NF-κB p65 protein expression.

### In-vivo, Mfn2 inhibits cervix carcinoma growth

**Fig. 3:**
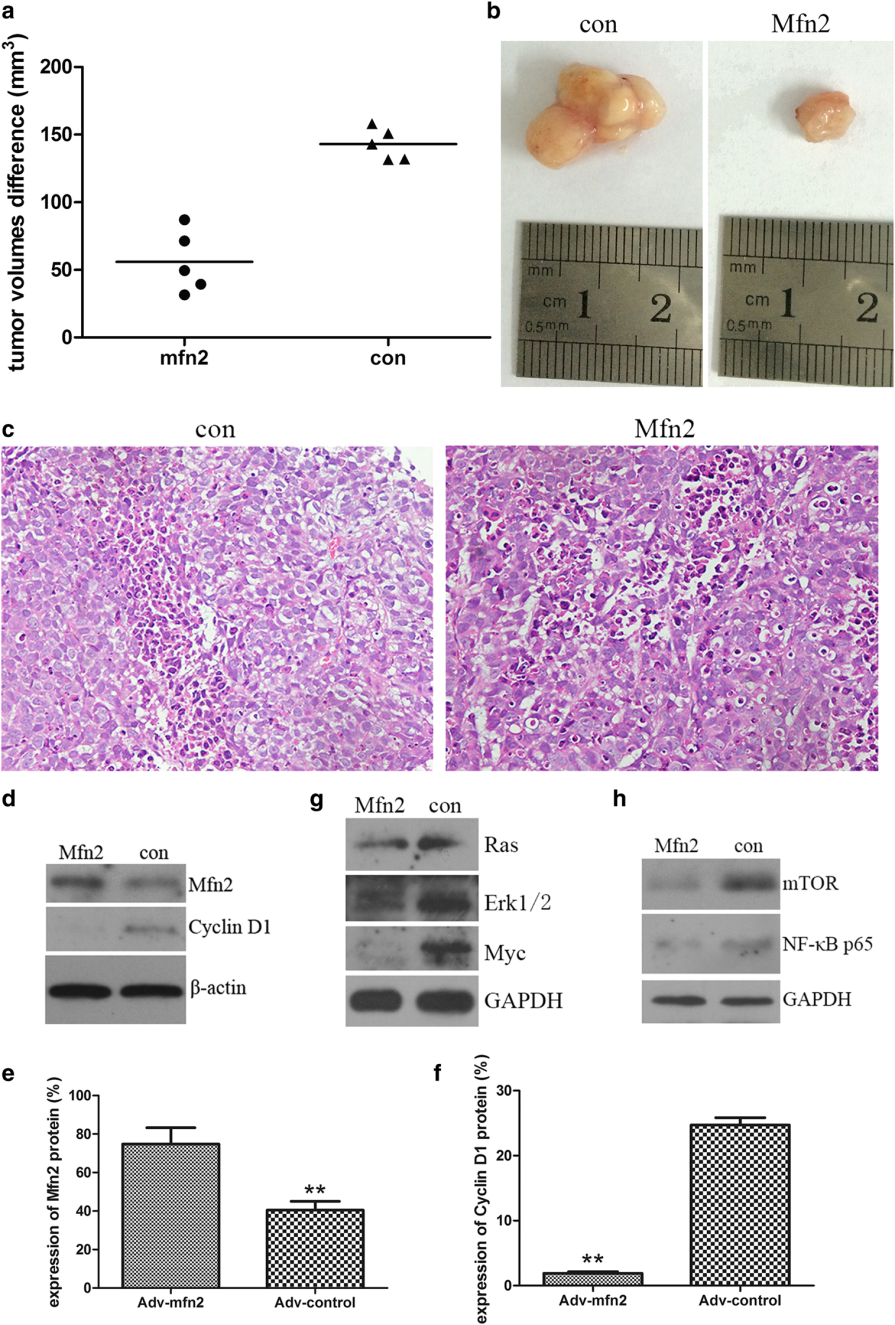
Mfn2 inhibited the tumor growth in vivo of cervical carcinoma mouse model and its signal pathway. **a** Determination of the tumor volume in mice treated with Adv-mfn2 or Adv-control for the 0, 3, 6 and 9 days. Data are represented as mean ± SD (**p < 0.01). **b** The sizes of xenografted cervix carcinoma after 14-days treatment in the Mfn2 group and the con group. **c** HE assay of tumor sections from animals treated with either Adv-control or Adv-Mfn2. Magnification: 200×. **d**–**f** The expression of Mfn2 and Cyclin D1 proteins in the tumors of mouse model was detected by western blot (**a**, **b**) and quantitation (**c**). Beta-actin was used as reference. Data are represented as mean ± SD (**p < 0.01). **g** Western blot showed Ras, Erk1/2 and Myc proteins expressions in cervix tumors treated with Adv-mfn2 after 2-weeks treatment. Total tumor tissue lysates were prepared and analyzed by Western blot for these proteins. GAPDH was used as reference. **h** Western blot showed mTOR and NF-κB proteins expressions in cervix tumors treated with Adv-mfn2 after 2-weeks treatment. Total tumor tissue lysates were prepared and analyzed by Western blot for these proteins. GAPDH was used as reference

In order to verify the effect of Mfn2 protein against the cervix carcinoma in vivo, we examined the therapeutic effect of Adv-mfn2 on xenografted cervix carcinoma in a mouse tumor model for a 2-week treatment. HeLa cells were injected subcutaneously and the animals were randomly divided into two groups of each 5 tumor-bearing mice. These tumor-bearing mice were treated with Adv-mfn2 or Adv-control as described in the Materials and Methods section. Before and after the treatment, the volume of the tumors was determined. During the treatment, the volume of xenografted cervix carcinomas treated with Adv-mfn2 increased slowly, whereas the volume of the tumors treated with Adv-control increased faster than the Adv-mfn2 group (Fig. 3a, b). At day 14, the mice were sacrificed and the tumors were macroscopically or histologically analyzed. The tumors treated with Adv-mfn2 were smaller than the ones treated with Adv-control (Fig. 3b). After the 2-week treatment all mice were checked, and there were not any metastases in the mice of two groups.

To detect the underlying mechanism of the Mfn2-triggered tumor reduction, tumor sections of both Adv-mfn2 and Adv-control treated tumors were analyzed by means of a HE assay and Western blot. The nucleus of cervix carcinoma cells treated with Adv-mfn2 was pycnosis and fragmented into several parts in the analyzed histological section. In contrast, the tumors treated with Adv-control had no signs of nucleus pycnosis (Fig. 3c). Then, Western blot was performed to confirm that Mfn2 was over expression in the tumor tissue of Adv-mfn2 group, and the expression of Cyclin D1 was decreased in Adv-control group (Fig. 3d–f). The expressions of Ras, Erk1/2, Myc, mTOR and NF-κB p65 protein in tumor tissues of 2 groups were detected by Western blot, and these were decreased by treated with Adv-mfn2 comparing with the Adv-control group (Fig. 3g, h). We found that Mfn2 could decrease the proteins of Ras-NF-κB signaling pathway.

## Discussion

In the current study, we confirmed that Mfn2 could inhibit Hela cells proliferation in vitro and in vivo, and arrest Hela cells cell-cycle. The cellular location of Mfn2 in Hela cells was performed by immunofluorescence. The results of CCK8 and the western blot of PCNA showed that Mfn2 inhibited the Hela cells proliferation in the dose- and time-dependence. The flow cytometry and the western blot of Cyclin D1 meant that the cell-cycle of Hela cells were arrested by Mfn2 in G0/G1 phase. It was been confirmed that Mfn2 was the inhibitor of Ras in VSMCs [15]. To find the signal pathway of cell-proliferation inhibition and cell-cycle arrest in Mfn2 manner in Hela cells, we detected the Ras, Myc, NF-κB p65, STAT3 proteins by western blot. The results of these western blots suggested that Mfn2 could inhibit the Hela cells by decreasing the expression of Ras and relative proteins in Ras-NF-κB signal pathway. In xenografted mouse model, we measured the tumor size before and after the Adv-mfn2 or Adv-control treatment, and analyzed the expression of Cyclin D1, Ras, Myc, ERK1/2, NF-κB p65, mTOR proteins by western blot. These results implicated that Mfn2 could inhibit the Hela cells growth by declining the expression of Ras protein and arresting the Ras-NF-κB signal pathway.

**Fig. 4:**
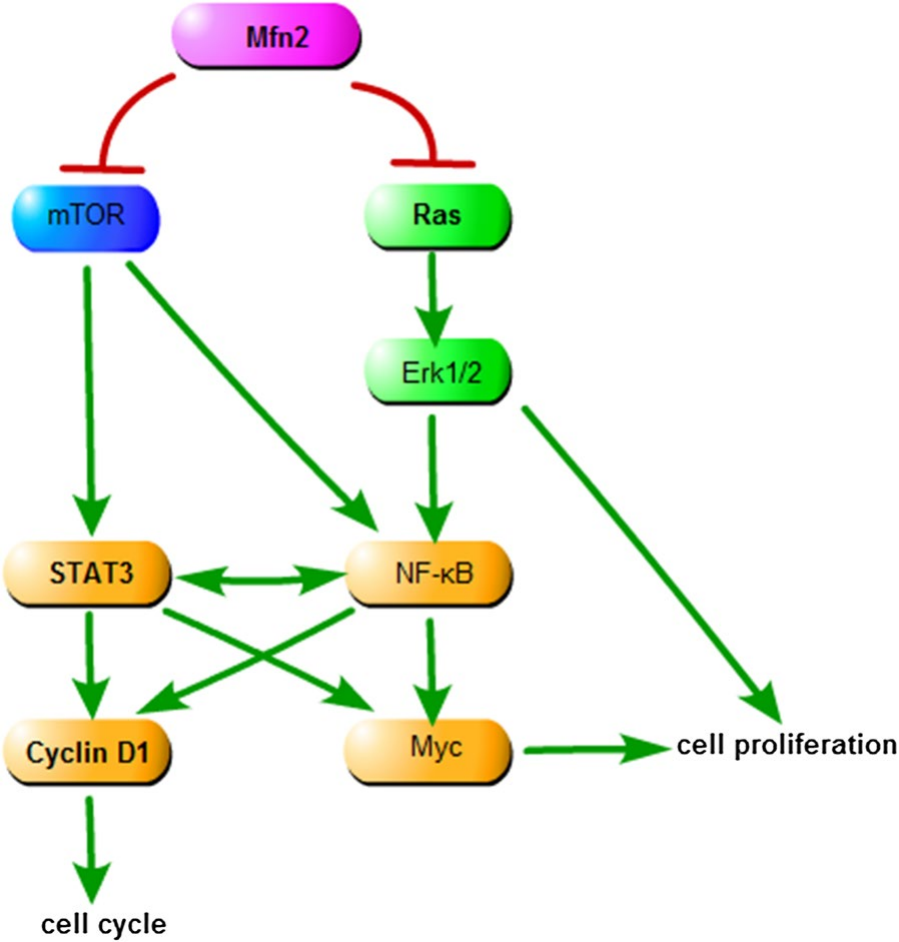
The network of Mfn2-Ras-NF-κB pathway in Hela cells

The proliferation of cells was remarkably inhibited, thus inducing the cells into apoptosis or senescence [23, 24]. In our previous study, the inhibition of Mfn2 in Hela cells has been proved to be achieved through the apoptosis induction [8] and cell cycle arrest. Mfn2 could inhibit Ras-Erk1/2 and PI3 k-Akt signal pathway in VSMCs [12, 15]. Ras-Erk1/2 and PI3k-Akt-mTOR are the classic signal pathways of cell proliferation [25, 26]. NF-κB has recently generated considerable interest as it has been implicated in human cancer initiation, progression and resistance to treatment [27]. Mutations of upstream signaling molecules, such as Ras, often lead to constitutive activation of NF-κB in solid malignancies [27, 28]. NF-κB could stimulate the transcription of proliferation regulating genes like Cyclin D1 and Myc [27, 29–31]. NF-kB does not function alone but is part of a network, which determines the pattern of its effects on the expression of STAT3 [32]. Meanwhile, the activation of STAT3 is responsible for genes that promote cell proliferation such as Cylinc D1, Myc and so on [33]. The activation of STAT3 could cause a positive feedback mechanism to NF-κB [34]. STAT3 and NF-κB work together in a network for a result [27]. Therefore, Mfn2 inhibits Ras- NF-κB signal pathway to arrest the cell proliferation and cell-cycle in Hela cells (Fig. 4). Based on our research, we propose that Mfn2 may be a novel target to the therapy of cervical carcinoma in the future.

## Conclusions

Mfn2 could inhibit the proliferation and cell cycle in Hela cells. The Ras-NF-κB signaling pathway was inactive by the expression of Mfn2 increasing. The xenografted cervical carcinoma mouse model was examined to confirm the effect of Mfn2 in Hela cells in vivo.

## Data Availability

Not applicable.

## Abbreviations

Mfn2: mitofusin 2
FBS: fetal bovine serum
CCK-8: Cell Counting Kit-8
